# *Plasmodium falciparum* genetic factors rather than host factors are likely to drive resistance to ACT in Ghana

**DOI:** 10.1186/s12936-020-03320-7

**Published:** 2020-07-15

**Authors:** Peter Hodoameda, Nancy Odurowah Duah-Quashie, Charles Oheneba Hagan, Sena Matrevi, Benjamin Abuaku, Kwadwo Koram, Neils Ben Quashie

**Affiliations:** 1 West African Center for Cell Biology of Infectious Pathogens, University of Ghana, P. O. Box LG54, Legon, Ghana; 2 Epidemiology Department, Noguchi Memorial Institute for Medical Research, College of Health Sciences, University of Ghana, P. O. Box LG581, Legon, Ghana; 3 Centre for Tropical Clinical Pharmacology and Therapeutics, University of Ghana Medical School, P. O. Box GP4236, Accra, Ghana; 4 University of Cape Coat Medical School, Cape Coast, Ghana

**Keywords:** Polymorphism, Mutation, Resistance, Cytochrome, Molecular markers, Prevalence

## Abstract

**Background:**

Artemisinin-based combination therapy (ACT) partner drugs, currently used in Ghana are lumefantrine, amodiaquine and piperaquine. *Plasmodium falciparum* isolates with reduced susceptibility to these partner drugs may affect treatment outcome. Mutations in *pfmdr1* gene is linked to reduced parasite susceptibility to amodiaquine and lumefantrine. In addition, the potency of the partner drugs in vivo depends on the metabolism by the cytochrome P450 (CYP) enzyme in the host. Mutations in the *CYP2C8* and *CYP3A4* genes are linked to reduced metabolism of amodiaquine and lumefantrine in vitro*,* respectively. This study investigated the host and parasite genetic factors affecting the susceptibility of the malaria parasite to ACT partner drugs.

**Methods:**

Archived samples from 240 patients age ≤ 9 years participating in anti-malarial drug resistance survey in Ghana, and given artemether with lumefantrine (AL) or artesunate with amodiaquine (AA), were selected and analysed. Polymerase chain reaction (PCR) followed by Sanger sequencing was used to determine the polymorphisms in *CYP2C8, CYP3A4* and *pfmdr1* genes.

**Results:**

For *CYP3A4*, all had wild type alleles, suggesting that the hosts are good metabolizers of lumefantrine. For *CYP2C8* 60% had wild type alleles, 35% heterozygous and 5% homozygous recessive alleles suggesting efficient metabolism of amodiaquine by the hosts. For *pfmdr1* gene, at codon 86, 95% were wild type (N86) and 5% mutant (Y86). For codon 184, 36% were wild type (Y184) and 64% mutant (F184) while for codons 1034, 1042 and 1246, 100% (all) were wild type. The high prevalence of N86-F184-D1246 haplotype (NFD) suggest presence of parasites with reduced susceptibility to lumefantrine and not amodiaquine. Delayed clearance was observed in individuals with mutations in the *pfmdr*1 gene and not cytochrome 450 gene. Both synonymous and non-synonymous mutations were observed in the *pfmdr*1 at low prevalence.

**Conclusion:**

The outcome of this study indicates that the parasite's genetic factors rather than the host’s are likely to drive resistance to ACT in Ghana.

## Background

Malaria caused by an infection of *Plasmodium falciparum* is one of the major causes of morbidity and mortality in sub-Saharan Africa, especially in children under 5 years old and pregnant women [1] The World Health Organization (WHO) recommends the use of a combination of a fast-acting artemisinin derivative and a relatively slow-acting partner drug, for the treatment of uncomplicated malaria in disease-endemic areas [2]. The recommended first-line artemisinin combination therapy (ACT) in Ghana for treating uncomplicated malaria is artesunate with amodiaquine (AA), artemether with lumefantrine (AL) or a combination of dihydroartemisinin with piperaquine [3]. The reason for combining the drugs (ACT) is to slow down the development of resistance to anti-malarial drugs by *P. falciparum* [4]. The fast-acting drug quickly reduces parasite load whilst the slow-acting anti-malarial gradually destroys residue parasites. The potency of ACT is dependent on the efficacy of both the artemisinin component and the partner drug [4]. Reduced susceptibility of parasites to partner drugs in ACT can potentially result in resistance to artemisinin in future as parasites that escape the fast action of artemisinin or its derivatives will not be cleared by the partner drug and this could allow for growth and expansion of a drug-resistant parasite population [4].

Variations observed in effectiveness of ACT in malaria-endemic regions are dependent on parasite genetic factors [5], as well as human genetic factors [6]. For parasite genetic factors, polymorphisms which arise due to single nucleotide changes in the *pfmdr1* gene in its coding region have been linked to differential parasite susceptibility to ACT partner drugs, such as amodiaquine [7] and lumefantrine [8]. This makes *pfmdr1* an important likely candidate for initiating ACT partner drug resistance [9].

The polymorphic *pfmdr1* alleles that are mostly found in Africa are N86Y, F184Y and D1246Y. The *P. falciparum* N86-F184-D1246 haplotype (NFD haplotype) has been linked to decreased susceptibility to anti-malarial drugs, such as mefloquine and lumefantrine. The selection of the NFD haplotype has been seen in malaria treatment using AL. The different haplotype, which is the Y86-Y184-Y1246 haplotype (YYY haplotype), is associated with reduced amodiaquine susceptibility [10].

Differences in genetic make-up of humans is the principal factor that defines the level of drug availability in the blood to clear the parasites [6]. The cytochrome P450 enzyme family (CYP genes) is involved in the metabolism of different anti-malarial drugs [6]. Amodiaquine is mainly metabolized by *CYP2C8* [11] whiles lumefantrine is metabolized mainly by *CYP3A4* [12]. Different mutations in the promoter region, introns or exons can result in different alleles of the CYP450 genes in different individuals. The metabolism of a drug or a combination of drugs could be decreased, increased or unaffected depending on the allele(s) an individual possesses [13]. Elucidating the exact role these disparities in the genes coding for the enzymes involved in ACT metabolism is vital for understanding the inter-individual pharmacokinetic differences observed in persons using ACT [14]. This study investigated *P. falciparum* and host genetic factors that are likely to affect the efficacy of ACT partner drugs used in Ghana.

## Methods

### Study design

Blood blot filter paper used in this study were archived samples collected in 2016 from children participating in studies previously described by Abuaku et al*.* [15]. The study of Abuaku et al. was part of routine surveillance on the therapeutic efficacy of ACT in Ghana; the efficacy of AA and AL were studied in six sentinel sites representing the forest and savannah zones of the country. Three sites representing the two ecological zones studied AA whilst the other three sites studied AL. Additionally, archived samples from a site in the Coastal savanna ecological zone was included in the analysis. In each site, the study was a one-arm, prospective evaluation of the clinical, parasitological and haematological responses to directly observed treatment with either AA or AL among children 6 months to 9 years old with uncomplicated falciparum malaria. The WHO 2009 protocol on surveillance of anti-malaria drug efficacy was used for the study with primary outcomes as prevalence of day 3 parasitaemia and clinical and parasitological cure rates on day 28.

An informed consent was obtained from each parent or guardian*.* A medical doctor prescribed either AA or AL to the study participants who were then followed up for 28 days.

**Fig. 1:**
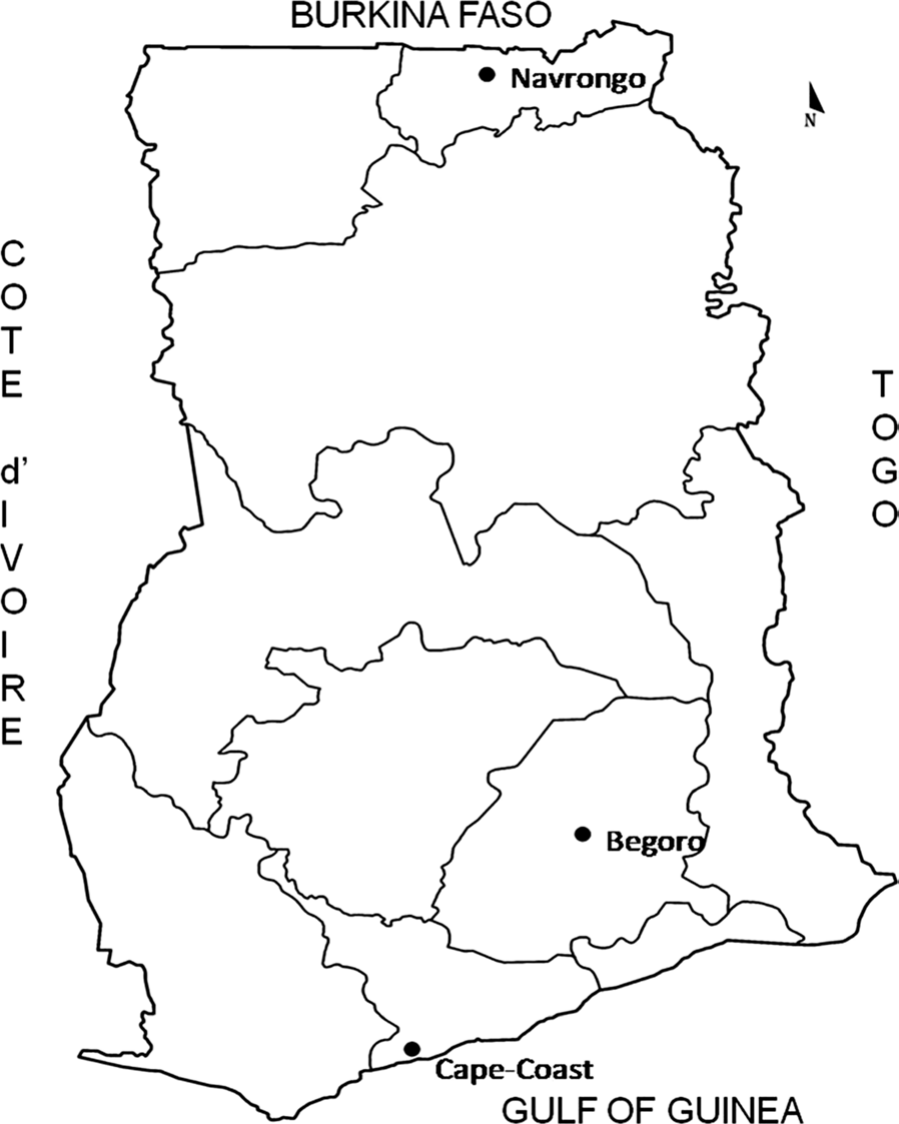
Map of Ghana showing location of study sites used in the study: Cape Coast, Begoro and Navrongo

The archived samples were selected from three sentinel sites, Navrongo, Begoro and Cape Coast, which represent three distinct eco-epidemiological zones in Ghana (Fig. 1). Begoro is located in the tropical forest ecological zone, Navrongo is in the northern savanna ecological zone and Cape Coast is situated in the coastal savanna ecological zone. The samples (100 μl blood) were collected on Whatman 3 filter paper (Sigma, UK), stored in plastic bags containing silica gel, and kept at room temperature until use.

The samples from a group of participants referred hereafter as ‘cohort 1′ comprised 120 recruited patients who were given AL, and a second group, cohort 2, comprised 120 patients who received AA. Of the 240 study participants’ archived samples analysed, 60 were selected from the savannah zone, 90 from the coastal zone and 90 from the forest zone.

### DNA extraction

Malaria parasite DNA was extracted from the archived blood blots filter papers using a QIAamp DNA minikit (Qiagen, Germany) following the manufacturer’s protocol. Convectional PCR [16] was performed to amplify the region of interest using published protocols [17–19]. PCR products were sequenced using Sanger sequencing at Macrogen, The Netherlands.

### Detection of *pfmdr1* polymorphisms

The regions of *pfmdr1* gene was amplified and sequenced to determine the presence of any mutation. The amplification was carried out using the protocol described by [17]. The polymorphisms were analysed at codons 86 (asparagine to tyrosine), 184 (tyrosine to phenylalanine), 1034 (serine to cysteine), 1042 (asparagine to aspartic acid) and 1246 (aspartic acid to tyrosine). A PCR followed by Sanger sequencing was used in determining these polymorphisms. Thirty microlitres aliquot of PCR products were kept on ice and shipped for sequencing at Macrogen, The Netherlands.

### Detection of *CYP2C8* and *CYP3A4* polymorphisms

The *CYP2C8* polymorphisms were analysed at codon 269 (isoleucine to phenylalanine). The *CYP3A4* polymorphisms were analysed at position *-392A* > *G* of the proximal promoter region. A PCR followed by Sanger sequencing was used to determine the polymorphism in *CYP2C8* as reported by Cavaco et al. [18] and *CYP3A4* as described by Hodel et al. [19]. A PCR product of 120 bp and 717 bp represents a successful amplification of *CYP2C8* and *CYP3A4*, respectively. Aliquot of the PCR products was shipped appropriately for sequencing at Macrogen, The Netherlands.

### Data analysis

Data were organized using R software, SPSS software (version 20) and GraphPad Prism version 6. Sequence data were analysed with the BLAST program (https://blast.ncbi.nlm.nih.gov/) to determine the authenticity of the sequences. Multiple sequences were aligned with MAFFT (EMBL.EBI, Hinxton, Cambridge, UK) using the 3D7 wild-type as reference. Consensus sequence editing and single nucleotide polymorphism (SNP) detection was carried out using the CLC Main Workbench 7.9.1 (Qiagen, Aarhus, Denmark). The *CYP2C8* sequences were aligned to *CYP2C8* (ENSG 00000138115) as reference sequence while *CYP3A4* sequences were aligned to *CYP3A4* (ENSG 00000160868) as reference sequence from NCBI database. Genotype deviations from the Hardy–Weinberg equilibrium were also determined. The Hardy–Weinberg equilibrium determines whether or not the allele or genotype frequencies for a particular gene will remain constant from generation to generation in the absence of evolutionary influences such as genetic drift, inbreeding and founder effect [20]. All tests in this study were considered statistically significant when *p* value < 0.005.

## Results

### Prevalence of individuals *CYP3A4* and *CYP2C8* genotype

**Fig. 2:**
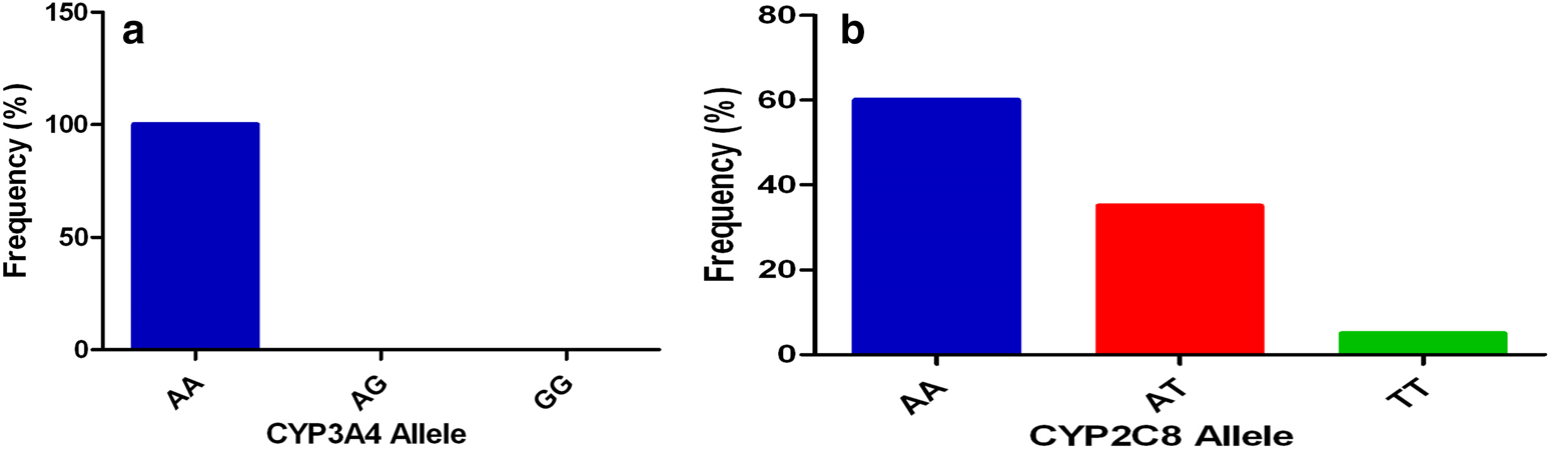
Prevalence of *CYP3A4* and *CYP2C8*.** a** Genotype for *CYP3A4*, AA is wild-type, AG is heterozygous, and GG is homozygous recessive; **b** Genotype for *CYP2C8*, AA is wild-type, AT is heterozygous, and TT is homozygous recessive

Ninety-three individuals were successfully genotyped for *CYP3A4* of which all (100%) were wild type (Fig. 2). The genotypes analysed for *CYP3A4* was not in Hardy–Weinberg equilibrium. Ninety-four individuals were successfully genotyped for *CYP2C8* of which 60% (56/94) had wild-type alleles, 35% (33/94) heterozygous and 5% (5/94) homozygous recessive alleles (Fig. 2). The genotypes analysed for *CYP2C8* was in Hardy–Weinberg equilibrium.

### Prevalence of isolates with *pfmdr*1 codons 86, 184, 1034, 1042, 1246 alleles and NFD haplotype

**Fig. 3:**
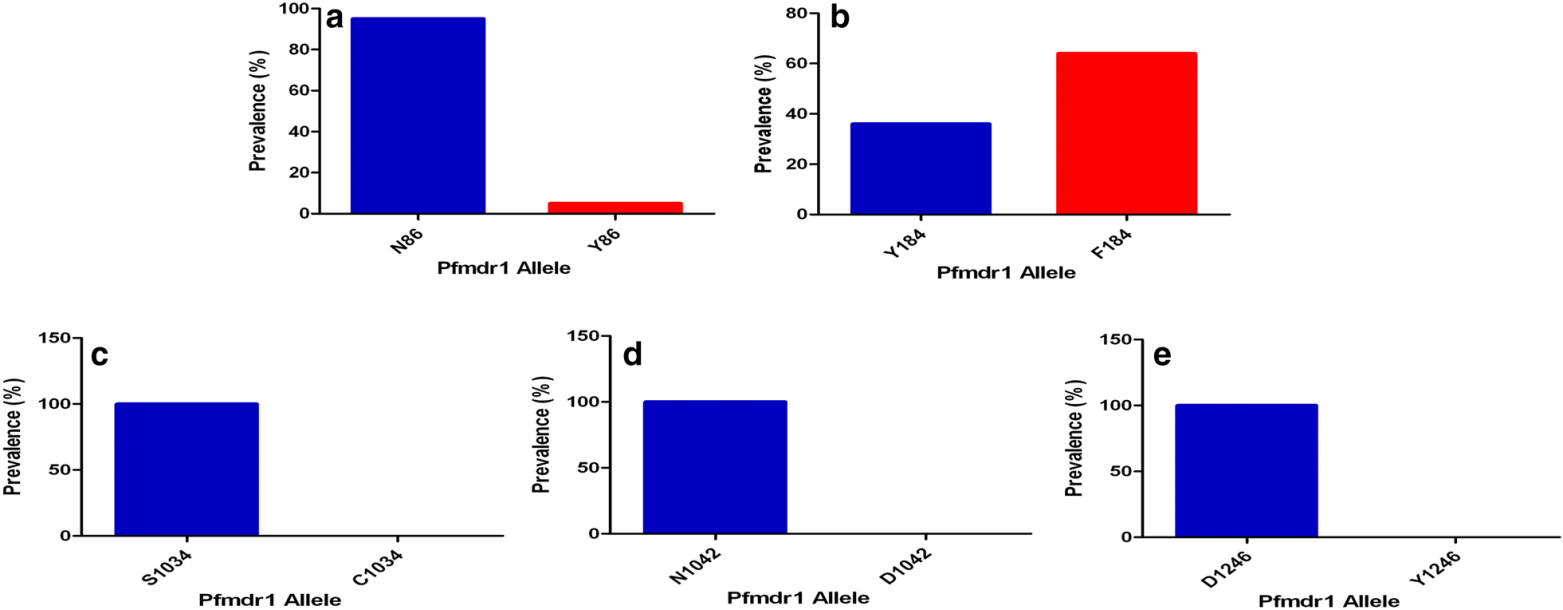
Prevalence of *pfmdr*1 codons 86, 184, 1034, 1042 and 1246. **a***pfmdr*1 86, **b***pfmdr*1 184, **c***pfmdr*11034, **d***pfmdr*1 1042 and **e***pfmdr*1 1246. The blue bar is for wild-type allele while the red bar is for the mutant allele

**Fig. 4:**
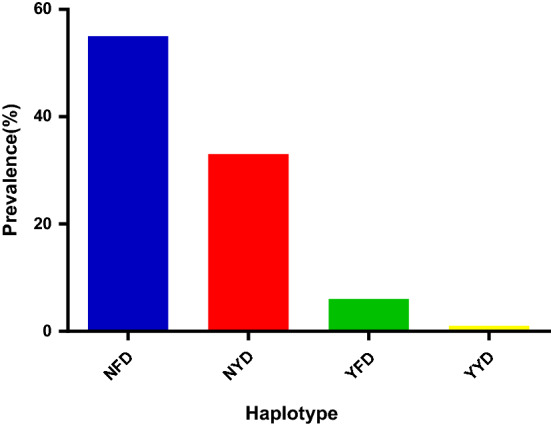
Prevalence of *pfmdr*1 haplotype**.** The NYD is the wild type haplotype while the NFD, YFD, and YYD are the mutant haplotypes

Ninety-five *P. falciparum* out of 120 clinical isolates was successfully genotyped for the *pfmdr*1 gene. For *pfmdr*1 genotype at codon 86, 95% (90/95) were wild-type (N86) and 5% (5/95) mutant (Y86) (Fig. 3). For codon 184, 36% (34/95) were wild-type (Y184), 64% (61/95) mutant (F184) while for codons 1034, 1042 and 1246, all (100%) were wild-type. There were both non-synonymous and synonymous mutations observed at low frequencies in some of the samples analysed (Table 1). The *pfmdr*1 haplotypes observed were 57.8% (55/95) NFD, 34.7% (33/95) NYD, 6.3% (6/95) YFD, and 1% (1/95) YYD (Fig. 4).

**Table 1:** Novel synonymous and non-synonymous mutations at coastal and savannah ecological zones

Ecological zone	Novel synonymous mutation [frequency in parenthesis]	Novel non-synonymous mutation [frequency in parenthesis]
Coastal	F106S [1], E236K [1], Y296N [1], E275K [1], E261K [1], S1217Y [1]	G293G [1], G102G [1], G284G [1], I1119I [1], L127L [1]
Savannah	E236K [1], E275K [3], R299K [2], E261K [3]	L108L [1], D117D [1], T1069T [1]

Non-synonymous mutations that lead to change in amino acid and synonymous mutations which do not lead to a change in amino acid, were both observed at low frequencies in some of the samples analysed. It must be noted that natural selection on both synonymous and non-synonymous mutations plays an important role in shaping levels of synonymous polymorphism. In this study, six novel synonymous mutations were observed in the coastal zone and 9 in the savannah zone. Multiply synonymous mutations were observed in samples from savannah zone. With regard to the novel non-synonymous mutations, 5 were observed in samples from the coastal zone whilst 3 were seen in samples from the savannah zone.

## Discussion

It is obvious that resistance of *P. falciparum* to ACT partner drugs may lead to the gradual evolution of strains of parasites with reduced susceptibility to artemisinin. The failure of partner drugs should therefore be of great concern to national malaria control programmes in disease-endemic areas. Since both the host and the parasite genome play a role in metabolism of ACT, a key question often asked is: what drives parasite resistance to ACT partner drugs? Before attempting to address this question, the clinical data generated in this study, which is a sub-set of data published elsewhere, must be examined. The clinical data indicate that 13% (16/120) of the participants treated with AL (cohort 1) still carried parasites on day 3 post-treatment, compared to 4% (5/120) of those given AA (cohort 2). However all parasites were cleared by day 7 post treatment. This indicates a better rate of parasite clearance with AA than AL. It is not surprising as this results gives credence to a previous report by Abuaku et al*.* [15]. The report indicate an overall PCR-corrected cure rate of 100% for AA and 97.6% (95% CI 93.1, 99.5) for AL: 97.2% (95% CI 92.0, 99.4) in the forest zone and 100% in the savannah zone [15]. The significantly high N86-F184-D146 haplotype in AL-treated individuals compared to Y84-Y184-Y1246 haplotype in AA-treated individuals observed here may explain the slight difference in efficacy between AL and AA observed in that study.

The efficacy of the partner drugs, amodiaquine and lumifantrine, investigated in this study, is linked to *pfmdr1* gene, which is part of the ATP-binding cassette (ABC) transporters [21]. This gene encodes a transporter which is found in the digestive vacuole of the parasite [22]. The *pfmdr1* is thought to function by pumping compounds out of the parasite, making it an important protein for anti-malarial drug resistance. The true mechanistic role of the *pfmdr1* in initiating anti-malarial drug resistance is poorly understood [9], although certain mutations in the gene have been associated with resistance to different anti-malarial drugs [7, 8]. The exact mechanism in which mutation at the *pfmdr1* F184 confers resistance to lumefantrine while mutations at the *pfmdr1* Y86 and Y1246 confer resistance to amodiaquine is not well understood but has been observed to be mostly selected during lumefantrine and amodiaquine drug pressure, respectively [7, 8].

There was high prevalence of N86, F184 and D1246 haplotypes in this study with no record of Y86, Y184 and Y1246 haplotypes. This observation is consistent with that reported by Duah et al. [23]. The results also show the widespread presence of these mutations in Ghana which are not ecological zonal bias. This was because there was no significant difference in these mutations across the different ecological zones (Additional file 1: Fig. S1 and Additional file 2: Fig. S2).

The cytochrome P450 enzyme family (CYP genes) is a key enzyme involved in the metabolism of different anti-malarial drugs [6]. Lumefantrine is metabolized to desbutyl-benflumetol mainly by *CYP3A4* [12]. A change from adenine (A) to guanine (G) at position 392 of *CYP3A4* gene proximal promoter region results in CYP3A4*1B allele [24].This mutant has been reported to have poor enzyme activity [25]. From the results obtained in the current study, 93 individuals were successfully genotyped for *CYP3A4* of which 100% had the wild-type gene. This observation suggests that lumefantrine is well metabolized in the participants. Again, delayed clearance observed in patients treated with AL was seen to have one or more mutations in the *pfmdr*1 gene of the *P. falciparum* clinical isolates rather than mutation in the *CYP3A4* gene of the individuals (Table 2). Based on these observations it can be strongly inferred that the parasite genetic factors could be the driving force behind drug efficiency in the children treated with AL, and this could possibly be the determinant of clinical resistance to the ACT in future. However, there is the need to tread cautiously since this inference is more or less speculative as it is not backed by any pharmacokinetic studies of desbutyl-lumefantrine in the children. It must however be emphasized that similar findings have been reported [26].

**Table 2:** *CYP3A4* wild-type individuals for lumefantrine metabolism and parasite *pfmdr*1 mutation(s) among those with delayed parasite clearance (day 3 positive)

Sample number	*CYP3A4* genotype	*pfmdr*1 mutation (s)
1	AA	F184
2	AA	F184
3	AA	F184
4	AA	F184
5	AA	F184
6	AA	F184
7	AA	F184
8	AA	Y86, F184
9	AA	F184
10	AA	F184
11	AA	F184
12	AA	F184
13	AA	F184
14	AA	F184
15	AA	F184
16	AA	F184

Sixteen of the samples with delayed clearance had the *CYP3A4* wild-type individuals for lumefantrine metabolism and parasite *pfmdr*1 mutation(s) as indicated

The *CYP2C8* is the main enzyme that metabolizes amodiaquine to desethyl amodiaquine (DEAQ) [27]. The wild-type CYP2C8*1 and the mutant CYP2C8*2 are the most predominant in Ghana [28]. A change from adenine (A) to thymine (T) at nucleotide position 895 on exon 5 results in the CYP2C8*2 mutant. CYP2C8*2 has been shown to be associated with decreased enzyme activity in vitro and reduced intrinsic clearance of amodiaquine [11]. From the results of the study, 94 individuals were successfully genotyped for *CYP2C8* of which 60% (56/94) had wild-type alleles, 35% (33/94) heterozygous and 5% (5/94) homozygous recessive alleles. This result is contrary to what has been reported by Kudzi et al., 2009 [28]. The high number of individuals with wild-type *CYP2C8* suggests that amodiaquine was well metabolized in the participants. However delayed clearance was observed in individuals who reported with high parasitaemia (> 100,000) on day 0 and with one or more mutation(s) in the *pfmdr1* gene. These individuals had either wild-type or heterozygous *CYP2C8* genotype (Table 3) suggesting ample concentration of DEAQ in their plasma. Thus it was expected that their parasites should have been easily cleared. There was no delayed clearance observed in CYP2C8*2 individuals. It is speculated that the *CYP2C8* genotype of an individual may not alter the metabolism of the drug significantly, hence the plasma concentration of DEAQ may be adequate to clear the parasite. The absence of delayed clearance in CYP2C8*2 individuals can also be explained by the fact that dihydroartemisinin (DHA), which is a metabolite of artesunate, clears most of the parasites and leaves only a few supposedly ‘weakened parasite’ residues making the presence of a sub-optimal concentration of DEAQ enough to clear the parasite residue in these individuals.

**Table 3:** *CYP2C8* wild-type and heterozygous individuals for amodiaquine metabolism and parasite *pfmdr*1 mutation(s) among those with delayed parasite clearance (day 3 positive)

Sample number	*CYP2C8* genotype	*pfmdr*1 mutation (s)
1	AA	F184
2	AA	F184
3	AT	F184
4	AT	Y86
5	AT	Y86, F184

When Chi square test was used to determine the association between *CYP2C8/ CYP3A4* and *pfmdr*1 genotypes and day 3 positivity, there was no significant difference. For the few cases of delayed parasite clearance using AA, the lack of association between the wild-type enzyme and the cases indicate that the host gene-type of the enzyme could not be responsible for the delayed parasite clearance. Therefore, this observation suggests that the parasite genetic factor among others could be responsible for the delayed clearance rather than the host genetic factors.

There were similar numbers of both non-synonymous and synonymous mutations observed at low frequencies in the coastal and forest ecological zones (Table 1). The synonymous mutations may not have any significant effect on the susceptibility of the parasite to the anti-malarial drugs since it does not lead to change in amino acids. However, the novel non-synonymous mutations observed in this study may suggest the possible emergence of new mutations that may lead to reduced parasite susceptibility to ACT in Ghana sooner than later.

## Conclusion

Observations made in this study give enough grounds to conclude that parasite genetic factors rather than the host’s is more likely to drive resistance to ACT, especially AL in Ghana. All individuals successfully genotyped for *CYP3A4* were wild-type, suggesting that lumefantrine is well metabolized in the participants. The high percentage of *CYP2C8* wild-type individuals also suggests that amodiaquine is metabolized efficiently. High prevalence of N86, F184 and D1246 suggests AL is less efficacious than AA. The outcome of this study conveys a warning that malaria parasites are becoming resistant to anti-malarial drugs. Prompt monitoring of ACT is required. There is the need to find other anti-malarial drugs that could be used as ACT partner drugs.

## Supplementary information

**Additional file 1: Figure S1** Distribution of *pfmdr1* codon 86 at the various ecological zones

**Additional file 2: Figure S2** Distribution of *pfmdr1* codon 184 at the various ecological zones

## Data Availability

All data generated or analysed during this study are included in this published article.

## Abbreviations

ACT: Artemisinin-based combination therapy
AA: Artesunate + amodiaquine
AL: Artemether + lumifantrine
CYP: Cytochrome
DEAQ: Desethyl amodiaquine
DHA: Dihydroartemisinin
*pfcrt*: *Plasmodium falciparum* chloroquine resistance transporter
*pfmdr1*: *Plasmodium falciparum* multidrug resistance 1
IRB: Institutional Review Board
NMIMR: Noguchi Memorial Institute for Medical Research
ABC: ATP-binding cassette
PCR: Polymerase chain reaction
WHO: World Health Organization
NFD: N86-F184-D1246 haplotype
YYY: Y86-Y184-Y1246 haplotype
