# Supplementary material for: Plasmodium falciparum genetic factors rather than host factors are likely to drive resistance to ACT in Ghana
Source: Malar J. 2020 Jul 15;19:255. doi: 10.1186/s12936-020-03320-7 (PMC7362516; doi:10.1186/s12936-020-03320-7)

**Supplementary Fig. 1 Distribution of *pfmdr1* codon 86 at the various ecological zones**

Kruskal-Wallis non-parametric test was carried to determine if there is any statistically significant difference between the locations. No difference was observed between the zones


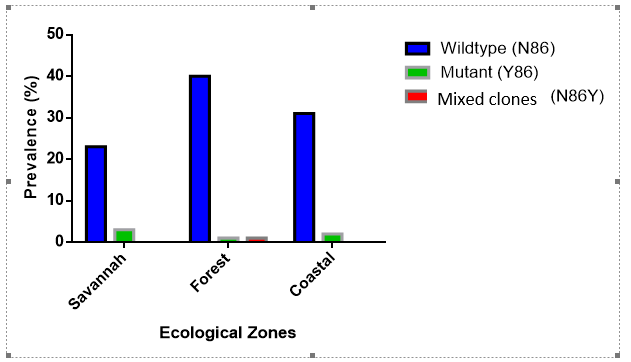

Supplement: Supplementary file 1 — Additional file 1: Figure S1 Distribution of pfmdr1 codon 86 at the various ecological zones [file 12936_2020_3320_MOESM1_ESM.docx]
