# Supplementary material for: Plasmodium falciparum genetic factors rather than host factors are likely to drive resistance to ACT in Ghana
Source: Malar J. 2020 Jul 15;19:255. doi: 10.1186/s12936-020-03320-7 (PMC7362516; doi:10.1186/s12936-020-03320-7)
Supplement: Supplementary file 2 — Additional file 2: Figure S2 Distribution of pfmdr1 codon 184 at the various ecological zones [file 12936_2020_3320_MOESM2_ESM.docx]

**Supplementary Fig. 2 Distribution of *pfmdr1* codon 184 at the various ecological zones**

Kruskal-Wallis non-parametric test was carried to determine if there is any statistically significant difference between the zones. No difference was observed.
